# Intratumoral Bacterial Profiles, Bacterial Co-Detection Patterns, and Molecular Alterations in Colorectal Cancer and Colorectal Polyps: A Vietnamese Cohort Study

**DOI:** 10.3390/microorganisms14102325

**Published:** 2026-10-12

**Authors:** Vu Son Giang, Ho Van Son, Dao Tien Manh, Nguyen Thanh Tung, Nguyen Phuong Thao, Nguyen Thi Than, Nguyen Dang Tung, Tran Viet Hao, Le Duy Cuong

**Affiliations:** 1 Diseases Department, Military Hospital 175, 786 Nguyen Kiem Street, Hanh Thong Ward, Ho Chi Minh City 700000, Vietnam; bsgiang175@gmail.com (V.S.G.); bsthaobv175@gmail.com (N.P.T.); 2 Department of Biochemistry, Military Hospital 175, 786 Nguyen Kiem Street, Hanh Thong Ward, Ho Chi Minh City 700000, Vietnam; hosonbioche175@gmail.com (H.V.S.); dstung175@gmail.com (N.T.T.); 3 Institute of Oncology and Nuclear Medicine, Military Hospital 175, 786 Nguyen Kiem Street, Hanh Thong Ward, Ho Chi Minh City 700000, Vietnam; dtmanh175@gmail.com; 4 Biochemistry Department, Military Hospital 175, 786 Nguyen Kiem Street, Hanh Thong Ward, Ho Chi Minh City 700000, Vietnam; nguyenthan289@gmail.com; 5 Department of Quality Management, 108 Military Central Hospital, 1st Tran Hung Dao Street, Hai Ba Trung Ward, Hanoi City 100000, Vietnam; dangtung108hqm@gmail.com; 6 Department of Experimental Medicine, 108 Military Central Hospital, 1st Tran Hung Dao Street, Hai Ba Trung Ward, Hanoi City 100000, Vietnam; drtranviethao243@gmail.com

**Keywords:** colorectal cancer, intratumoral microbiota, bacterial colonization, microbial co-colonization patterns

## Abstract

Intratumoral microbiota may contribute to colorectal cancer (CRC), but their relationships with bacterial co-occurrence, relative bacterial loads, and molecular alterations remain incompletely understood. This study characterized intratumoral bacterial profiles in CRC and colorectal polyps and examined their clinicopathological and molecular associations. A cross-sectional study included 149 patients with CRC and 109 patients with benign colorectal polyps. *F. nucleatum*, ETBF, *A. muciniphila*, and *eae*^+^ *E. coli* were detected in formalin-fixed, paraffin-embedded tissues using multiplex real-time PCR, with relative bacterial loads quantified using the 2^−ΔCt^ method. *KRAS* and *BRAF* V600E mutations and *HSP110ΔE9* transcripts were assessed by real-time PCR/RT–qPCR. After multivariable adjustment for age, sex, tumor location, and the other bacterial species, ETBF and *eae*^+^ *E. coli* were independently associated with CRC (adjusted ORs, 4.69 and 6.26, respectively; both *p* < 0.001). Relative loads of all four bacterial species were higher in CRC than in polyps (*p* < 0.001 for all comparisons). ETBF + *eae*^+^ *E. coli* showed the strongest dual-species association with CRC (adjusted OR, 10.64; 95% CI, 4.49–25.24), while *F. nucleatum* + *A. muciniphila* + *eae*^+^ *E. coli* showed the strongest triple-species association (adjusted OR, 21.56; 95% CI, 5.02–92.60). Increasing numbers of detected bacterial species were positively associated with CRC (*p* for trend < 0.001). CRC was characterized by higher relative bacterial loads and more interconnected bacterial co-colonization patterns than colorectal polyps. These findings support a community-level association between intratumoral bacterial profiles and CRC and warrant validation in larger multicenter longitudinal studies.

## 1. Introduction

Colorectal cancer (CRC) is one of the most common malignancies worldwide and remains a major global public health challenge. According to the latest GLOBOCAN estimates, more than 2.0 million new cases of CRC and approximately 918,000 deaths were estimated to occur worldwide in 2024. CRC ranks as the third most commonly diagnosed cancer and the leading cause of cancer-related mortality globally [1]. The substantial global burden of CRC highlights the continuing need for effective strategies for prevention, early detection, and treatment. In Vietnam, CRC has also become an important public health burden. According to the International Agency for Research on Cancer (2024) [2], 22,584 new CRC cases were estimated, accounting for approximately 12.7% of all newly diagnosed cancers, while 10,990 deaths were attributed to CRC, representing approximately 9.9% of all cancer deaths. CRC ranked third in cancer incidence and fourth in cancer mortality in Vietnam in 2024 [2]. Because early CRC is frequently asymptomatic or presents with nonspecific clinical manifestations, many patients are diagnosed at advanced stages. Consequently, survival outcomes decline substantially with disease progression, ranging from approximately 90% 5-year survival for localized disease to only 14% for patients with distant metastatic disease who are not eligible for curative surgical treatment [3].

Increasing evidence suggests that tumor-associated bacteria originating from the intestinal microbiota play an important role in colorectal carcinogenesis. Microbial dysbiosis and intratumoral bacterial colonization may contribute to tumor initiation and progression through multiple mechanisms, including induction of genomic instability, activation of oncogenic signaling pathways, disruption of epithelial barrier integrity, modulation of immune responses, and alteration of the tumor microenvironment. Furthermore, bacterial metabolites and toxin-producing microorganisms can induce DNA damage and contribute to intestinal tumorigenesis [4].

Among bacterial species associated with CRC, *Fusobacterium nucleatum* (*F. nucleatum*) has been the most extensively investigated and is considered a potential microbial driver of colorectal carcinogenesis through activation of β-catenin signaling, suppression of antitumor immune responses, and promotion of chronic inflammation [5]. *Bacteroides fragilis* (*B. fragilis*), particularly enterotoxigenic strains, produces *B. fragilis* toxin, which can disrupt epithelial barrier integrity and activate NF-κB and STAT3 signaling pathways, thereby promoting inflammatory responses and tumor development [6]. *Akkermansia muciniphila* (*A. muciniphila*) is generally recognized as a beneficial mucin-degrading bacterium involved in maintaining intestinal barrier function and immune homeostasis; however, its role in CRC appears to be context-dependent, with both potentially protective and tumor-promoting effects reported [7]. Certain pathogenic strains of *Escherichia coli* (*E. coli*), particularly those carrying the pks genomic island, produce the genotoxin colibactin, which induces DNA double-strand breaks, genomic instability, and mutational events associated with colorectal carcinogenesis [8,9]. Collectively, these findings indicate that different bacterial species may contribute to CRC development through distinct but potentially complementary biological mechanisms [10,11,12].

In parallel, molecular alterations involving the genes *KRAS*, *BRAF*, and *HSP110* are important determinants of colorectal tumor biology and have implications for prognosis and therapeutic decision-making [13,14]. Activating mutations in *KRAS* and *BRAF* lead to constitutive activation of the MAPK signaling pathway, resulting in uncontrolled cell proliferation and resistance to anti-EGFR therapies [13,15]. Alterations in *HSP110*, particularly the HSP110ΔE9 splicing variant observed in microsatellite instability (MSI)-associated colorectal cancers, have been associated with tumor characteristics and therapeutic response [14]. Emerging evidence indicates that intratumoral bacteria may interact with host genomic alterations, oncogenic signaling pathways, and the tumor immune microenvironment; however, the relationships between specific bacterial colonization patterns and molecular alterations remain incompletely understood [16,17].

Although the role of intestinal microbiota in CRC has been increasingly recognized, most previous studies have focused on individual bacterial species, fecal microbiota composition, or populations from Western countries [10,11,12,16]. Comprehensive studies simultaneously evaluating multiple intratumoral bacterial species together with their associations with clinicopathological characteristics and clinically relevant molecular alterations remain limited, particularly in Southeast Asian populations [17,18]. Consequently, the intratumoral distribution of multiple bacterial species and their relationships with clinicopathological features and clinically relevant molecular alterations remain insufficiently characterized, particularly in Southeast Asian populations [17,19]. Comparative studies of bacterial profiles between CRC and benign colorectal polyps are also scarce, especially at the intratumoral level [20].

Therefore, this study aimed to characterize intratumoral bacterial signatures of *F. nucleatum*, *enterotoxigenic Bacteroides fragilis* (ETBF), *A. muciniphila*, and *eae*-positive *E. coli* (*eae^+^ E. coli*) in CRC and benign colorectal polyps and to examine their associations with clinicopathological characteristics, co-colonization patterns, bacterial burden, and molecular alterations in *KRAS*, *BRAF*, and HSP110ΔE9. By integrating microbial and molecular analyses in a Vietnamese cohort, this study provides insights into the differences in intratumoral bacterial profiles between CRC and benign colorectal polyps and their associations with clinically relevant molecular features.

## 2. Materials and Methods

### 2.1. Sample Preparation

A cross-sectional study was conducted using a consecutive sampling method. All potentially eligible patients who underwent surgical resection for CRC or endoscopic polypectomy for colorectal polyps at the Department of Gastrointestinal Surgery, 175 Military Hospital, Ho Chi Minh City, Vietnam, between May 2023 and December 2024 were consecutively considered for inclusion.

All patients with CRC and colorectal polyps underwent the same bowel preparation protocol before tissue collection. No prophylactic antibiotics were administered before tissue collection. A total of 294 cases were initially screened, including 172 CRC cases and 122 colorectal polyp cases. After application of the predefined eligibility criteria, 23 CRC cases were excluded because of prior antibiotic/probiotic use within 1 month before tissue collection and/or other chronic inflammatory bowel diseases, while 13 polyp cases were excluded because of prior antibiotic/probiotic use within 1 month before sampling and/or histopathological evidence of dysplasia or malignancy. The final sample comprised 149 CRC and 109 colorectal polyp cases (total *n* = 258).

Histopathological diagnoses were established according to the World Health Organization (WHO) classification by experienced pathologists.

Formalin-fixed, paraffin-embedded (FFPE) tissue blocks were retrieved from the Department of Pathology. Representative tumor-rich areas containing more than 70% tumor cells were identified and marked by an experienced pathologist. The selected FFPE blocks were then sectioned into 5-μm-thick slices using a microtome and stored at 4 °C until DNA extraction. To minimize cross-contamination, the microtome and working surfaces were thoroughly cleaned, and tissue debris was removed between specimens. Blank paraffin sections were processed alongside the study specimens.

#### Inclusion and Exclusion Criteria

CRC group. Patients aged ≥18 years with histologically confirmed primary colorectal adenocarcinoma who had not received chemotherapy or radiotherapy before tissue collection were eligible for inclusion. Patients were excluded if they had received antibiotics, immunosuppressive agents, or probiotics within one month before the date of tissue collection; had a history of other malignancies; had chronic inflammatory bowel diseases (e.g., Crohn’s disease or ulcerative colitis) or other chronic inflammatory diseases affecting the colorectum; or had incomplete clinical or pathological records.

Polyp group. Patients aged ≥18 years with histologically confirmed benign colorectal polyps without epithelial dysplasia who underwent endoscopic polypectomy and provided written informed consent were eligible for inclusion. Patients were excluded if they had used antibiotics, immunosuppressive agents, or probiotics within one month before sample collection; had histopathological evidence of epithelial dysplasia or malignancy; or had incomplete clinical or pathological records.

For both groups, specimens with insufficient tissue, insufficient DNA yield, or poor DNA quality for molecular analyses were excluded.

Clinicopathological characteristics, including tumor location, histological subtype, tumor grade, and TNM stage, were retrieved from pathological reports. Tumor stage was classified according to the American Joint Committee on Cancer (AJCC) Cancer Staging Manual (8th edition) [21], whereas histopathological classification was based on WHO Classification of Tumors: Digestive System Tumors (5th edition) [22].

### 2.2. Histopathological Examination

FFPE tissue specimens were processed using standard histopathological procedures. Tissue samples were fixed in 10% neutral buffered formalin, dehydrated through graded ethanol, cleared in xylene, and embedded in paraffin. Paraffin blocks were sectioned into 3–4 μm-thick slices using a rotary microtome and mounted on glass slides. After drying at 56 °C for 1 h, the sections were stained with hematoxylin and eosin (H&E) for routine histopathological evaluation. Histopathological diagnoses were established according to the WHO Classification of Tumors of the Digestive System by two experienced pathologists who were blinded to the molecular findings. Histological subtype, tumor differentiation grade, and the presence or absence of epithelial dysplasia in colorectal polyps were assessed before molecular analyses.

### 2.3. DNA Extraction

Genomic DNA was extracted from FFPE tissue sections using the QIAamp DNA FFPE Tissue Kit (Qiagen, Hilden, Germany) according to the manufacturer’s instructions. Briefly, FFPE tissue sections were deparaffinized, followed by tissue lysis with proteinase K and DNA purification using silica membrane spin columns. Extraction blanks were included in each extraction batch and processed alongside the study specimens. The extracted DNA was used for subsequent real-time polymerase chain reaction (real-time PCR) analyses to detect *F. nucleatum*, ETBF, *A. muciniphila*, *eae^+^ E. coli*, and mutations in the *KRAS*, *BRAF*, and *HSP110* genes. DNA samples were stored at −20 °C until molecular analysis.

### 2.4. RNA Extraction

Total RNA was extracted using the RecoverAll™ Total Nucleic Acid Isolation Kit for FFPE (Invitrogen, Carlsbad, CA, USA) according to the manufacturer’s instructions. Following genomic DNA extraction, the remaining cellular pellet from each FFPE sample was retained for total RNA isolation. The extracted RNA was subsequently used for reverse transcription real-time polymerase chain reaction (RT–qPCR) analysis and stored at −80 °C until use.

### 2.5. Bacterial Detection Using Multiplex Real-Time PCR Assay

Primer and probe sets were selected or redesigned based on previously published assays and target gene sequences retrieved from the GenBank database (National Center for Biotechnology Information, NCBI). Target sequences were evaluated by sequence alignment, and primer/probe compatibility was assessed using the OligoAnalyzer™ Tool (Integrated DNA Technologies, IDT, Coralville, IA, USA; https://www.idtdna.com/pages/tools/oligoanalyzer) and NCBI Primer-BLAST (National Center for Biotechnology Information, Bethesda, MD, USA; https://www.ncbi.nlm.nih.gov/tools/primer-blast/). For redesigned assays, conserved target regions were selected based on available reference sequences. Oligonucleotides were synthesized by Integrated DNA Technologies (IDT, Singapore). The target genes included *bft-1* for ETBF (GenBank accession No. AB026625.1), *nusG* for *F. nucleatum*, *RluA* for *A. muciniphila*, and *eae* for *eae*+ *E. coli*. The human *SLCO2A1* gene (GenBank accession No. NG_031964.3) was used as an internal control to assess the quality of input DNA. *SLCO2A1* (also known as prostaglandin transporter, PGT) was selected as the human genomic DNA internal control because it is a single-copy autosomal gene that has been used as a reference target for normalization of bacterial DNA in colorectal tissue, including FFPE specimens [23]. The primer and probe sequences are presented in Table 1, with additional information on their sources and reference sequences provided in Supplementary Table S1.

DNA concentrations were normalized to 30 ng/μL before amplification. Multiplex real-time PCR was performed using an ABI 7500 Real-Time PCR System (Applied Biosystems, Singapore). Two multiplex reactions were used: Reaction 1 simultaneously detected ETBF (*bft-1*) and *F. nucleatum* (*nusG*), whereas Reaction 2 detected *A. muciniphila* (*RluA*), *eae*^+^ *E. coli* (*eae*), and human *SLCO2A1*. Each reaction was performed in a final volume of 30 μL and contained 150 ng of template DNA. In Reaction 1, the final concentrations of the *bft-1* and *nusG* primers were 200 nM each, with corresponding probe concentrations of 100 nM. In Reaction 2, the final concentrations of the *RluA* and *SLCO2A1* primers were 200 nM each, whereas the *eae* primers were used at 150 nM; probe concentrations were 100 nM for *RluA* and *SLCO2A1* and 75 nM for *eae*. The complete reaction composition and reagent volumes are provided in Supplementary Table S1. Recombinant plasmids containing the corresponding target sequences were included as positive controls, and no-template controls were included in each run.

The amplification protocol consisted of an initial denaturation at 95 °C for 2 min, followed by 45 cycles of 95 °C for 30 s and 60 °C for 30 s, with fluorescence acquisition at 60 °C. The *SLCO2A1* Ct value was first assessed to verify the quantity and amplifiability of human genomic DNA. Samples with an *SLCO2A1* Ct >34.059 or without detectable amplification were re-extracted or re-analyzed.

Target-specific Ct thresholds were established from plasmid standard curves. Ct values below 36.602 for *bft-1*, 36.297 for *nusG*, 34.791 for *RluA*, and 34.408 for *eae* were considered positive for ETBF, *F. nucleatum*, *A. muciniphila*, and *eae*^+^ *E. coli*, respectively. Ct values at or above the corresponding thresholds, or absence of amplification, were considered negative. Relative bacterial abundance was calculated using the 2^−ΔCt^ method, where ΔCt = Ct(target) − Ct(*SLCO2A1*).

Analytical performance was evaluated using recombinant plasmid standards prepared from cloned target fragments. The amplicons were purified, cloned, and verified by Sanger sequencing before plasmid copy numbers were calculated from plasmid concentration and size. Ten-fold serial dilutions ranging from 10^6^ to 10 copies per reaction were tested in 13 independent replicates. Five standard curves were generated for *bft-1*, *nusG*, *RluA*, *eae*, and *SLCO2A1*. The corresponding standard-curve characteristics, including slope, amplification efficiency, and coefficient of determination (R^2^), are summarized in Supplementary Table S2, while the mean Ct values and replicate numbers at each plasmid concentration are provided in Supplementary Table S3. All assays showed sigmoidal amplification curves, high linearity (R^2^ = 0.9995–1.0000), and amplification efficiencies of 94.37–100.55%. All five targets were detectable at 10 copies per reaction, which represented the lowest concentration tested and detected.

**Figure 1:**
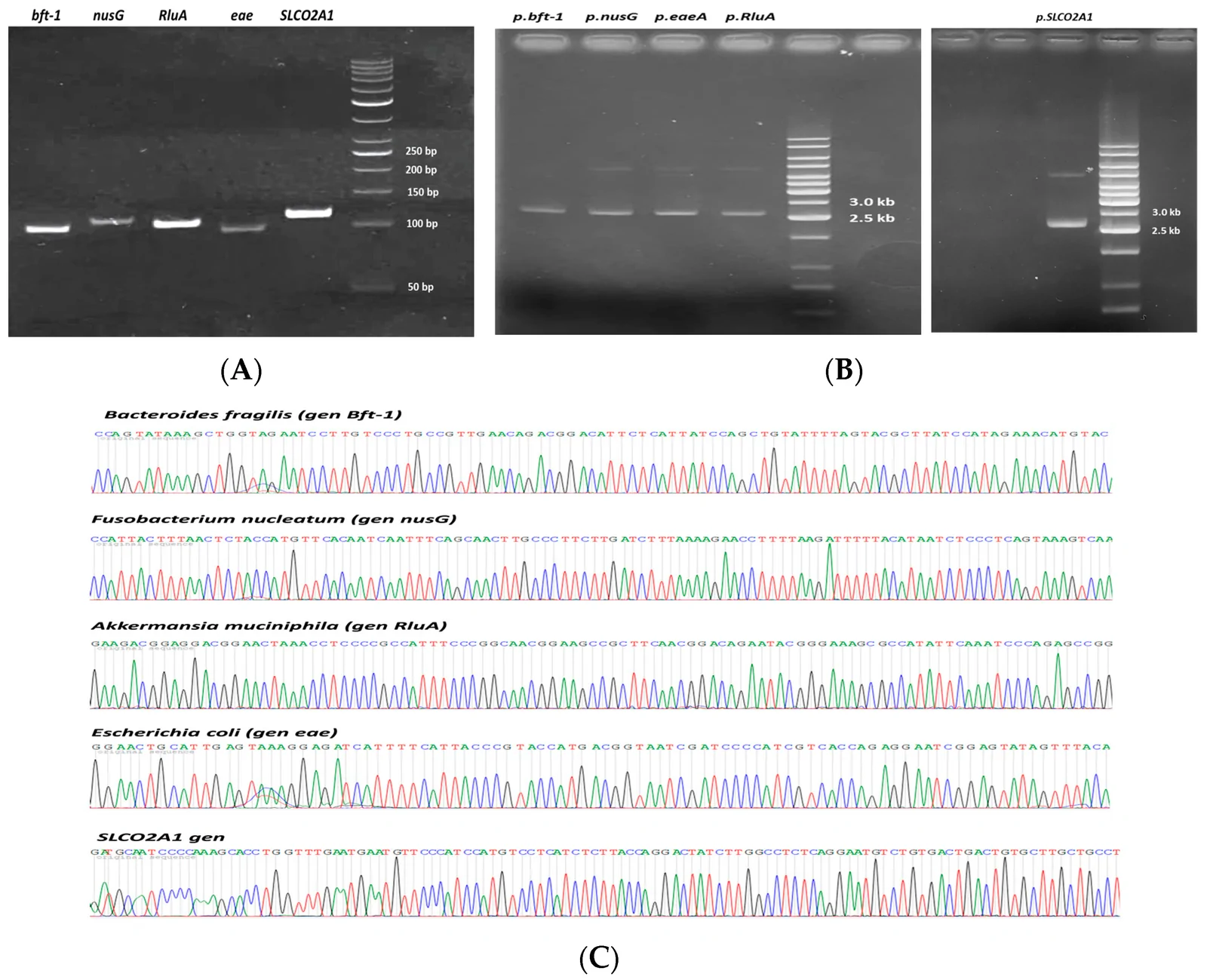
Molecular validation of bacterial and host gene targets by PCR, plasmid analysis, and Sanger sequencing. (**A**) Purified PCR products containing the *bft-1, nusG, RluA, eae*, and *SLCO2A1* target sequences. (**B**) Extracted plasmids containing the corresponding inserted fragments. (**C**) Sanger sequencing results confirming the inserted *bft-1, nusG, RluA, eae*, and *SLCO2A1* fragments, corresponding to ETBF, *F. nucleatum*, *A. muciniphila*, *eae^+^ E. coli*, and the human *SLCO2A1* gene, respectively.

Analytical specificity was assessed in silico using sequence alignment and NCBI Primer-BLAST. The identity of the amplified target fragments was experimentally confirmed by PCR amplification, cloning, and Sanger sequencing (Figure 1). A formal cross-reactivity assessment using a predefined panel of non-target organisms was not performed. Formal limits of detection (LoD) and limits of quantification (LoQ) were not established using predefined statistical acceptance criteria; therefore, 10 copies per reaction is reported only as the lowest concentration tested and detected, rather than as a formal LoD or LoQ.

### 2.6. Detection of BRAF, KRAS, and HSP110 Alterations by Real-Time PCR

#### 2.6.1. Detection of BRAF Mutations

*BRAF* mutations were analyzed using the AmoyDx^®^ *BRAF* V600 Mutations Detection Kit (Amoy Diagnostics Co., Ltd., Xiamen, China) according to the manufacturer’s instructions. The assay detects six *BRAF* V600 mutations (V600E, V600K, V600E2, V600R, V600D, and V600D2) using the Amplification Refractory Mutation System (ARMS) combined with real-time PCR. Allele-specific primers selectively amplify mutant DNA, while mutation-specific fluorescent probes enable real-time detection. According to the manufacturer, the assay is capable of detecting mutant alleles present at approximately 1% in a background of wild-type DNA. Amplification was performed on an ABI 7500 Real-Time PCR System (Applied Biosystems, Thermo Fisher Scientific, Singapore), and mutation status was interpreted according to the manufacturer’s quality control criteria.

#### 2.6.2. Detection of KRAS Mutations

*KRAS* mutations were detected using the AmoyDx***^®^*** *KRAS*/*NRAS* Mutations Detection Kit (Amoy Diagnostics Co., Ltd., Xiamen, China) according to the manufacturer***’***s instructions. The assay detects 19 *KRAS* mutations (eight in exon 2, six in exon 3, and five in exon 4), together with 13 *NRAS* mutations located in exons 2, 3, and 4, using ARMS-based real-time PCR. Allele-specific primers and mutation-specific fluorescent probes enable selective amplification and real-time detection of mutant alleles. An external control supplied with the kit was used to assess DNA quality. According to the manufacturer’s specifications, the assay has an analytical sensitivity of approximately 1% mutant DNA in a background of wild-type DNA. Amplification was performed on an ABI 7500 Real-Time PCR System (Applied Biosystems, Thermo Fisher Scientific, Singapore).

#### 2.6.3. Detection of the HSP110ΔE9 Transcript by Real-Time RT-PCR

The HSP110ΔE9 transcript was detected by real-time reverse transcription PCR (RT–qPCR) using total RNA extracted from FFPE tissue specimens. Two primers were used to amplify the target transcript: HSP110F (5′-AGATATTAGCACAACACTCAATGC-3′), located in exon 8, and HSP110R (5′-GAGCAGCATGGTTTCGACTAA-3′), located in exon 10. Two hydrolysis probes were designed to distinguish the wild-type and exon 9 deletion transcripts: Probe R1 (5′-FAM-AGTATTGCACACTGTAATGCACATCCTCTG-3′), spanning the exon 8–9 junction, and Probe R2 (5′-HEX-CTCATGAACACTGTAATGCACATCCTCTG-3′), spanning the exon 8–10 junction.

RT–qPCR was performed under optimized reaction conditions on an ABI 7500 Real-Time PCR System (Applied Biosystems, Thermo Fisher Scientific, Singapore). Each run included two positive controls: a recombinant plasmid containing the wild-type *HSP110* transcript (WT_HSP110_E9) and a recombinant plasmid containing the *HSP110ΔE9* transcript (Delta_HSP110_truncate_E9). Samples with Ct values higher than those of the wild-type control were reanalyzed using increased RNA input. Samples that did not meet the predefined quality criteria after repeat testing were excluded from subsequent analyses. Relative HSP110ΔE9 expression was calculated using the 2^−ΔCt^ method 2^(CtWT−CtΔE9)^. Samples with a relative expression value ≥ 0.5 were considered positive for the HSP110ΔE9 transcript.

### 2.7. Statistical Analysis

Statistical analyses were performed using SPSS Statistics version 26.0 (IBM Corp., Armonk, NY, USA) and R version 4.5.2 (R Foundation for Statistical Computing, Vienna, Austria). R was used to generate the forest plots and bacterial co-occurrence networks. Continuous variables are presented as mean ± standard deviation (SD) or median (interquartile range [Q1–Q3]), as appropriate, and categorical variables as frequencies and percentages. Continuous variables were compared using the independent-samples *t*-test or Mann–Whitney *U* test, as appropriate, and categorical variables using Pearson’s chi-square or Fisher’s exact test.

Associations between bacterial detection and CRC status were assessed using logistic regression, with crude and adjusted odds ratios (ORs) and 95% confidence intervals (CIs) estimated from univariable and multivariable models, respectively. Models for CRC status were adjusted for age, sex, and tumor location, whereas analyses of clinicopathological and molecular characteristics within the CRC group were adjusted for age and sex. Binary and multinomial logistic regression were used for dichotomous and multicategory outcomes, respectively. Firth’s penalized logistic regression was used to estimate adjusted ORs and 95% CIs in the presence of complete or quasi-complete separation.

The number of detected bacterial species was analyzed both categorically, using samples with no detected bacteria as the reference, and continuously. Pairwise associations between bacterial species were assessed using the phi (φ) coefficient, with *p*-values adjusted using the Benjamini–Hochberg false discovery rate (FDR) procedure. Bacterial loads according to molecular alteration status were compared using the Mann–Whitney *U* test. All tests were two-sided, with *p* < 0.05 considered statistically significant unless otherwise specified.

Network density was calculated as the proportion of significant edges among all possible pairwise connections: D = E/[N(N − 1)/2], where *E* is the number of significant edges and *N* is the number of nodes. Edges represent statistically significant associations after FDR correction.

## 3. Results

As shown in Table 2, the CRC and polyp groups were comparable in age (adjusted *p* = 0.505). After Benjamini–Hochberg correction, male sex was more prevalent in the polyp group than in the CRC group (83.5% vs. 68.5%; adjusted *p* = 0.024), whereas rectal lesions were more common in CRC than in polyps (44.3% vs. 18.4%; adjusted *p* = 0.030). Adenomatous polyps were the predominant histopathological type (91/109, 83.5%), followed by hyperplastic polyps (15/109, 13.8%), whereas Peutz–Jeghers polyps were the least common (1/109, 0.9%).

Overall, any *KRAS* mutations were significantly more prevalent in CRC than in polyps (36.9% vs. 21.1%; adjusted *p* = 0.024). No significant differences were observed for *BRAF* mutation or other *KRAS* subtypes. In contrast, HSP110ΔE9 transcripts were significantly more frequent in polyps than in CRC (37.6% vs. 17.4%; adjusted *p* = 0.004).

Table 3 shows that among the 149 CRC patients, conventional adenocarcinoma was the predominant histological subtype (77.9%), and exophytic tumors were the most common macroscopic type (59.7%). Most patients presented with advanced local invasion (T4: 66.4%) and regional lymph node metastasis (N1–N3: 89.9%), while distant metastasis was observed in 10.1% of cases. Moderately differentiated tumors were predominant (88.6%).

As shown in Table 4, ETBF and *eae*^+^ *E. coli* were significantly associated with CRC status after multivariable adjustment, with adjusted ORs of 4.69 (95% CI: 2.41–9.13) and 6.26 (95% CI: 2.92–13.42), respectively (both *p* < 0.001). *F. nucleatum* was significantly associated with CRC in the univariable analysis (*p* = 0.001), but this association was no longer significant after multivariable adjustment for age, sex, tumor location, and the presence of the other bacterial species (*p* = 0.342). Similarly, *A. muciniphila* was not significantly associated with CRC after adjustment (*p* = 0.351).

**Figure 2:**
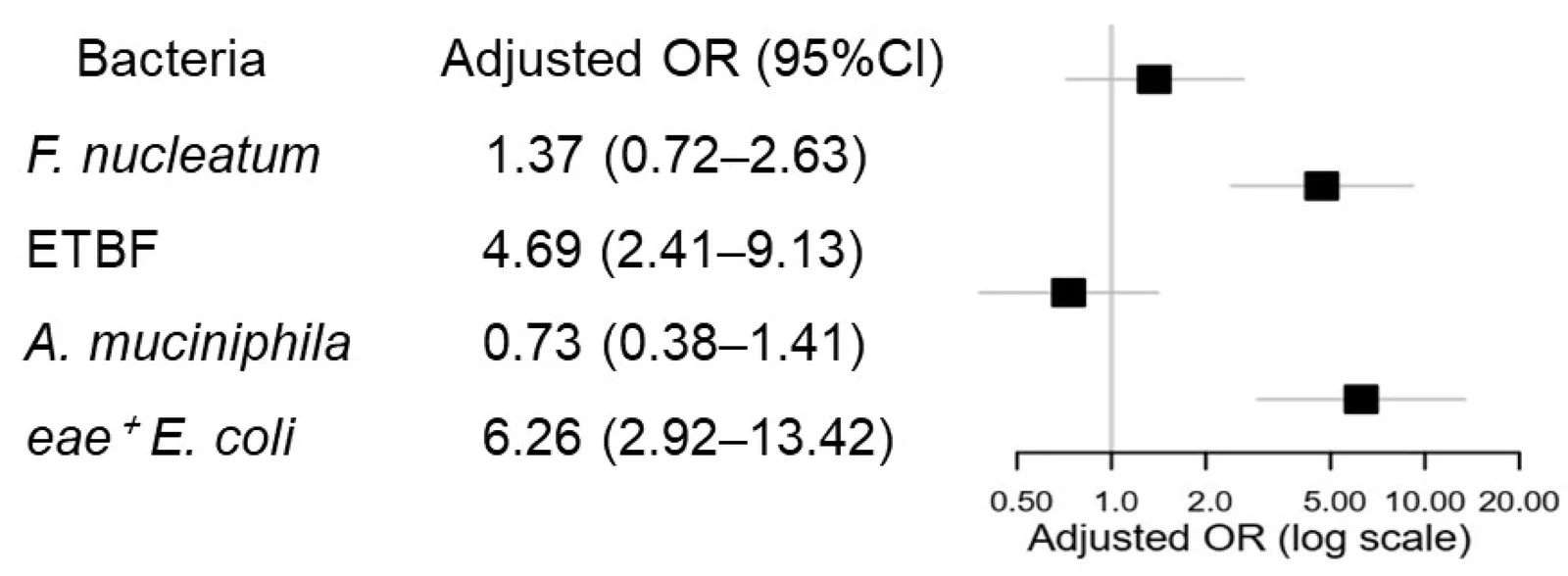
Forest plot of adjusted ORs for the association between intratumoral bacterial colonization and CRC status.

As shown in Figure 2, ETBF and *eae^+^ E. coli* remained significantly associated with CRC status after multivariable adjustment, whereas the associations for *F. nucleatum* and *A. muciniphila* were not statistically significant after adjustment. These findings indicate that the associations between individual intratumoral bacterial species and CRC status differed after accounting for potential confounding factors.

**Figure 3:**
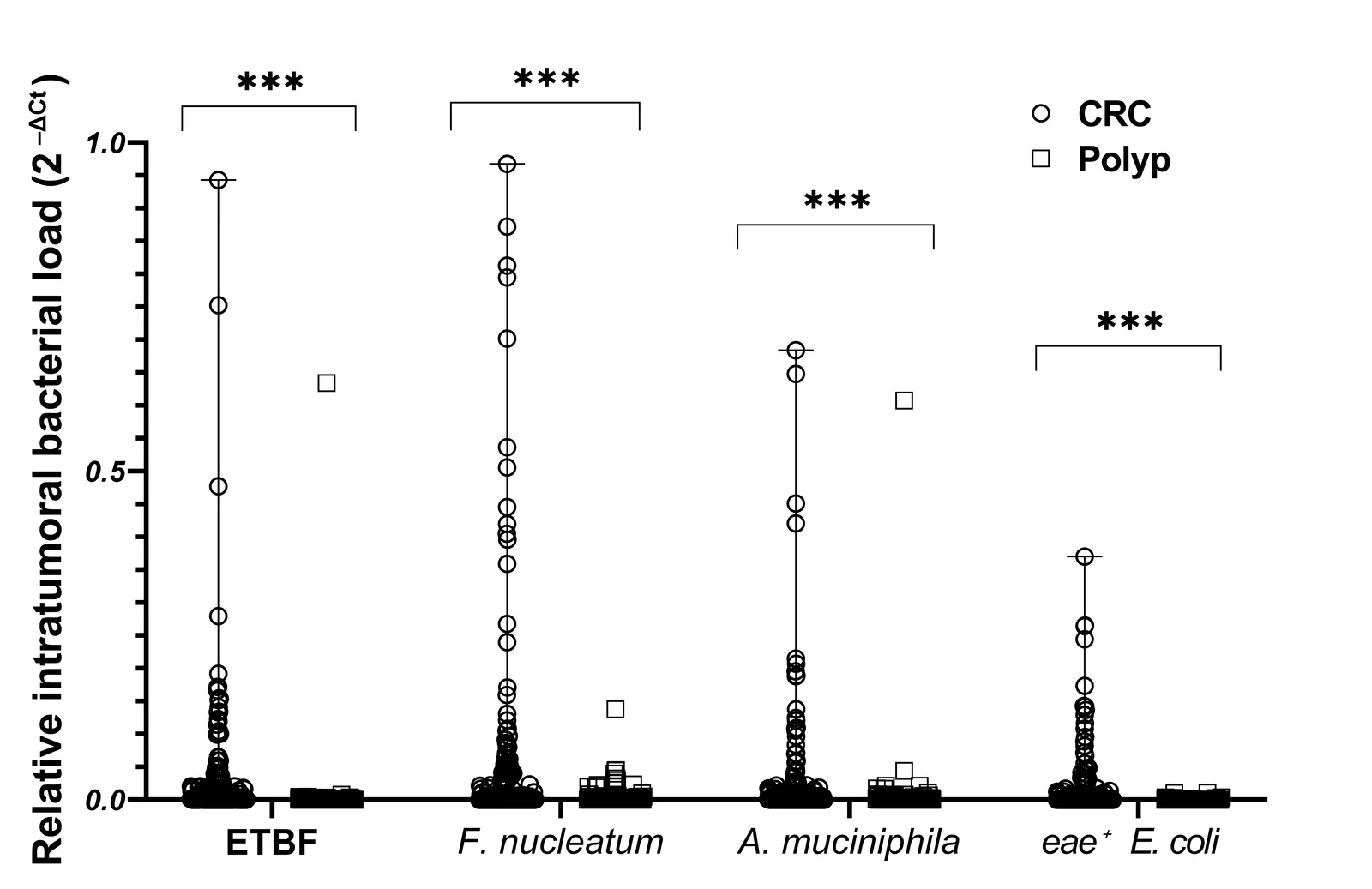
Distribution of intratumoral bacterial load (2^−ΔCt^) according to bacterial species in CRC and colorectal polyps. Note: Data are presented as individual values. *p-*values were determined using the Mann–Whitney U test. *** p < 0.001.

As shown in Figure 3, the relative intratumoral bacterial loads of all four bacterial species were significantly higher in CRC tissues than in colorectal polyps (*p* < 0.001 for all comparisons). The distributions were highly skewed, with most samples showing low bacterial loads and a smaller proportion exhibiting markedly higher values. These findings indicate that intratumoral bacterial load was generally elevated in CRC compared with colorectal polyps across all four bacterial species examined.

Table 5 shows that multivariable logistic regression analysis demonstrated that intratumoral bacterial co-occurrence patterns were significantly associated with colorectal cancer after adjustment for age, sex, and tumor location. All estimable bacterial co-occurrence patterns remained statistically significant after Benjamini–Hochberg FDR correction (adjusted *p* < 0.001). Among dual-species patterns, ETBF + *eae^+^ E. coli* co-occurrence had the strongest association with CRC (adjusted OR = 10.64, 95% CI: 4.49–25.24). Among triple-species patterns, the strongest association was observed for *F. nucleatum* + *A. muciniphila* + *eae^+^ E. coli* co-detection (adjusted OR = 21.56, 95% CI: 5.02–92.60). Co-detection with all four bacterial species was detected exclusively in CRC tissues and was absent in colorectal polyps; therefore, the corresponding odds ratio was not estimable because of complete separation. These findings indicate that more complex bacterial co-occurrence patterns were more frequently observed in CRC tissues.

As shown in Table 6, the distribution of intratumoral bacterial load differed significantly between CRC and polyp groups (*p* for trend < 0.001). Compared with tumors with no detected bacterial species, the adjusted odds of CRC were 0.67 (95% CI, 0.27–1.66; *p* = 0.392), 2.06 (95% CI, 0.91–4.79; *p* = 0.084), and 3.84 (95% CI, 1.73–8.86; *p* < 0.001) for tumors harboring one, two, and three bacterial species, respectively. Four-species co-detection was observed in 36 CRC cases (24.2%) but in no polyp cases. Because of complete separation, Firth’s penalized logistic regression was used, yielding an adjusted OR of 112.00 (95% CI, 13.71–14,614.13; *p* < 0.001). The wide confidence interval indicates limited precision of this estimate.

**Figure 4:**
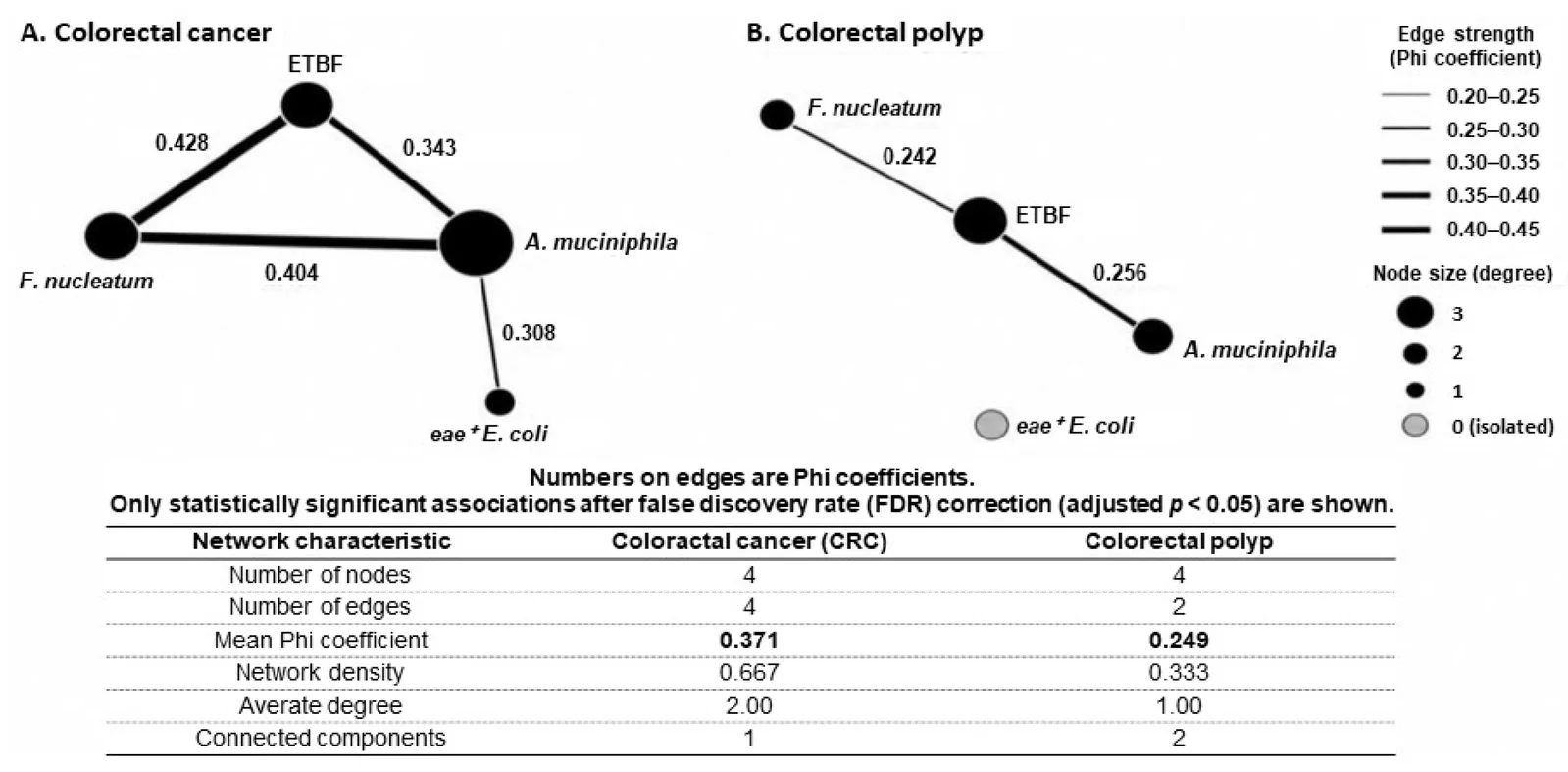
Differential bacterial co-occurrence networks in CRC and colorectal polyps. Note: Only statistically significant associations after FDR correction (adjusted *p*< 0.05) are shown.

As shown in Figure 4, Nodes represent bacterial species, and edges indicate statistically significant pairwise microbial associations after FDR correction (adjusted p < 0.05), measured using the Phi coefficient. Edge thickness is proportional to the Phi coefficient, while node size represents the degree (number of significant connections). The colorectal cancer network exhibits greater connectivity (4 edges; density = 0.667; mean Phi = 0.371) than the colorectal polyp network (2 edges; density = 0.333; mean Phi = 0.249). *eae^+^ E. coli* appears as an isolated node in the polyp network because no statistically significant associations with other bacterial species were detected.

Table 7 shows that, after adjustment for age and sex and correction for multiple comparisons using the Benjamini–Hochberg FDR procedure, none of the examined clinicopathological characteristics were significantly associated with intratumoral colonization by ETBF, *F. nucleatum*, *A. muciniphila*, or *eae^+^ E. coli* (all adjusted *p* > 0.05). Although *F. nucleatum* colonization was associated with ulcerative–exophytic tumors before FDR correction (adjusted OR, 5.10; 95% CI, 1.42–18.33; *p* = 0.013), this association was not significant after multiple-comparison correction (adjusted *p* = 0.143).

Associations between individual intratumoral bacterial detection and molecular alterations are presented in Table 8. After adjustment for age and sex and correction for multiple comparisons using the Benjamini–Hochberg FDR procedure, none of the examined bacterial species was significantly associated with *KRAS* mutation, *BRAF* V600E mutation, or HSP110ΔE9 transcript expression (all adjusted *p* > 0.05). An exploratory association was observed between *F. nucleatum* detection and *KRAS* exon 3 mutations before FDR correction (adjusted OR = 13.68, 95% CI: 1.38–135.89; *p* = 0.026); however, this association did not remain statistically significant after multiple-comparison correction (adjusted *p* = 0.130). The wide 95% CI further indicates limited precision of the effect estimate.

Table 9 shows that no significant association was observed between intratumoral bacterial detection and *KRAS* mutation status after adjustment for age, sex, and tumor location. In the continuous-variable model, the number of detected bacterial species was not significantly associated with the odds of *KRAS* mutation (adjusted OR = 1.01, 95% CI: 0.73–1.41; *p* for trend = 0.944). Associations between bacterial detection and *BRAF* V600E mutation or HSP110ΔE9 transcript status could not be estimated because of insufficient events and complete separation.

Intratumoral bacterial loads according to molecular alterations in CRC are shown in Table 10. Among the evaluated bacterial species, ETBF load was higher in tumors harboring *KRAS* mutations than in *KRAS* wild-type tumors (median [Q1–Q3]: 0.01 [0.00–0.10] vs. 0.00 [0.00–0.02]; nominal *p* = 0.037); however, this difference was not statistically significant after Benjamini–Hochberg FDR correction (adjusted *p* = 0.444). No significant differences were observed in the loads of *F. nucleatum*, *A. muciniphila*, or *eae^+^ E. coli* according to *KRAS* mutation status after FDR correction. Similarly, none of the evaluated bacterial loads were significantly associated with *BRAF* V600E mutation or HSP110ΔE9 transcript expression after multiple-comparison correction.

## 4. Discussion

The present study provides a comprehensive evaluation of intratumoral bacterial communities in CRC by assessing bacterial presence, microbial co-colonization patterns, bacterial burden, and molecular alterations within the same Vietnamese cohort. Unlike most previous studies that investigated only individual bacterial species or fecal microbiota, our study simultaneously examined intratumoral microbial signatures together with clinically relevant molecular alterations, thereby providing a more comprehensive characterization of the CRC microbial ecosystem. Increasing evidence suggests that intratumoral microbiota constitute an important component of the tumor microenvironment [24,25] and actively contribute to tumor initiation [24,26], immune modulation, genomic instability, and therapeutic responses [25,26,27] rather than representing passive colonizers.

One of the major findings of our study was that ETBF remained independently associated with CRC after multivariable adjustment (*p* < 0.001). This finding is biologically plausible, particularly in the context of ETBF, which produces *B. fragilis* toxin (BFT), a zinc-dependent metalloprotease that disrupts epithelial barrier integrity and activates pro-inflammatory and pro-tumorigenic signaling pathways, including NF-κB and STAT3. ETBF-associated signaling can further promote Th17/IL-17-mediated inflammation, epithelial proliferation, and a tumor-promoting microenvironment [27]. Our findings are consistent with the large Australian molecular study by Joo et al. (2024), which identified intratumoral ETBF in colorectal tumor tissues and investigated its associations with clinicopathological and molecular characteristics in 1697 tumor-derived DNA samples [17]. These findings are also consistent with a recent systematic review and meta-analysis reporting a higher prevalence of ETBF detection among patients with CRC than among healthy controls [28].

Similarly, *eae*^+^ *E. coli* was independently associated with CRC in our cohort. Although our assay targeted *eae^+^ E. coli* rather than pks^+^ *E. coli*, this finding is consistent with accumulating evidence that certain pathogenic *E. coli* strains, particularly genotoxic pks^+^ strains, can contribute to colorectal carcinogenesis [29,30]. These strains produce colibactin, a genotoxic metabolite that induces DNA damage, including DNA double-strand breaks and genomic instability, and is associated with a characteristic mutational signature, SBS88, in colorectal tumors [8,30,31]. Colibactin-producing *E. coli* has also been shown to promote epithelial injury and tumor development through direct interactions with intestinal epithelial cells, supporting a mechanistic link between bacterial colonization and colorectal carcinogenesis [32]. Moreover, epidemiological evidence indicates substantial geographical variation in exposure to colibactin-producing *E. coli*, suggesting that the contribution of these genotoxic bacteria to CRC may vary across populations [33]. Genomic and experimental studies have additionally demonstrated that colibactin-producing *E. coli* can induce characteristic mutational signatures in colorectal cancer, providing further mechanistic evidence linking these bacteria to colorectal carcinogenesis [30,31]. Thus, the independent association between *E. coli* colonization and CRC observed in our Vietnamese cohort adds to the growing evidence linking *E. coli*-associated microbial exposures with colorectal carcinogenesis across geographically diverse populations.

Interestingly, although *F. nucleatum* showed a higher prevalence in CRC tissues than in colorectal polyps, its independent association disappeared after adjustment for the presence of other bacterial species. This finding suggests that *F. nucleatum* may exert its tumor-promoting effects at least partly within a polymicrobial community rather than as an isolated carcinogenic microorganism. Recent spatial and microbiological studies have demonstrated that colorectal tumor biofilms are predominantly polymicrobial and frequently contain *F. nucleatum*, supporting the importance of microbial community interactions in the tumor microenvironment [34]. *F. nucleatum* can also interact with other tumor-associated bacteria, including *B. fragilis*, within biofilm-like niches, potentially reinforcing inflammatory and immunosuppressive signaling [35]. In parallel, *F. nucleatum* has been shown to modulate oncogenic pathways, including β-catenin signaling, while contributing to immune evasion and epithelial–mesenchymal transition [35,36]. Taken together, these findings and our results support the concept that the biological significance of *F. nucleatum* may depend partly on its microbial context and interactions with other tumor-associated bacteria.

A further important finding was the strong association between bacterial co-occurrence patterns and CRC. Several dual- and triple-species combinations were strongly associated with CRC, with ETBF + *eae*^+^ *E. coli* showing the strongest association among dual-species patterns (adjusted OR = 10.64, 95% CI: 4.49–25.24), and *F. nucleatum* + *A. muciniphila* + *eae*^+^ *E. coli* showing the strongest association among triple-species patterns (adjusted OR = 21.56, 95% CI: 5.02–92.60). All four bacterial species were simultaneously detected in 24.2% of CRC tissues but in none of the colorectal polyps; this complete separation was addressed using Firth’s penalized logistic regression, which yielded a finite adjusted OR of 112.00 (95% CI: 13.71–14,614.13; *p* < 0.001), although the wide confidence interval indicates limited precision and warrants cautious interpretation. These findings indicate that complex co-occurrence patterns are markedly enriched in CRC and provide additional evidence that tumor-associated bacteria may occur as interconnected microbial communities rather than as isolated organisms [34,37,38,39]. This community-level interpretation is consistent with recent reviews emphasizing the ecological nature of the intratumoral microbiota and the potential importance of interspecies interactions in shaping the tumor microenvironment [37,38].

Consistent with this pattern, the number of detected bacterial species was positively associated with CRC, with a significant trend across increasing bacterial species counts (*p* for trend < 0.001). Although the association for two detected species did not reach statistical significance after Firth correction (*p* = 0.084), the overall trend remained significant. Rather than implying a causal dose–response relationship, this pattern suggests that increasing bacterial species co-occurrence may reflect greater microbial complexity within CRC tissues [37,38,40].

The quantitative and network analyses further supported the distinct microbial profiles of CRC and colorectal polyps. Relative loads of all four bacterial species were significantly higher in CRC tissues than in colorectal polyps (*p* < 0.001 for all comparisons), although the distributions were highly skewed and showed substantial intertumoral variability [38,41]. The bacterial co-detection network was also more interconnected in CRC, with greater network density and mean Phi coefficient. Notably, *eae*^+^ *E. coli* showed no significant associations in the polyp network but was integrated into the more connected CRC network, suggesting that microbial relationships may be reorganized in the tumor environment. Together, these findings indicate that CRC-associated microbiota differ from those of colorectal polyps in both bacterial abundance and community structure, consistent with evidence of structured polymicrobial communities in CRC-associated biofilms [42].

Collectively, our findings have several important clinical implications. Rather than relying on a single bacterial biomarker, integrated microbial signatures incorporating bacterial abundance, bacterial burden, and bacterial co-detection networks may provide a more comprehensive basis for CRC risk stratification and early detection. Moreover, as intratumoral microbiota are increasingly recognized as modulators of chemotherapy and immunotherapy efficacy, characterization of tumor-associated microbial ecosystems may facilitate future precision medicine approaches.

### Limitations

This study has several limitations. First, its cross-sectional design precludes establishing causal or temporal relationships between intratumoral bacterial detection and CRC status. Second, the study used consecutive sampling in a single tertiary hospital in Vietnam, which may limit generalizability to other populations and healthcare settings; as a non-probability sampling approach, potential selection bias cannot be completely excluded. Third, some molecular alterations, particularly *BRAF* V600E and HSP110ΔE9, had relatively few positive cases, resulting in unstable estimates or complete separation in multivariable analyses. Finally, bacterial detection and quantification were based on DNA extracted from FFPE tissues and therefore cannot distinguish viable bacteria from non-viable or extracellular DNA or determine their spatial localization and functional activity. Future multicenter, longitudinal studies incorporating RNA-based viability assays, quantitative microbial profiling, and spatial and functional analyses are warranted to validate these findings and further elucidate intratumoral microbial–tumor interactions.

## 5. Conclusions

This study demonstrates that intratumoral bacterial profiles differ significantly between CRC and colorectal polyps, with higher relative bacterial loads and more extensive co-occurrence patterns observed in CRC tissues. ETBF and *eae*^+^ *E. coli* were independently associated with CRC, and increasing numbers of detected bacterial species were also positively associated with CRC. Overall, these findings support a community-level association between intratumoral bacterial profiles and CRC and highlight the potential importance of bacterial co-occurrence and microbial complexity in the tumor microenvironment.

## Figures and Tables

**Table 1:** Primers and probes used for bacterial detection and internal control.

Target	Primer/Probe	Sequence (5′-3′)	Amplicon Size (bp)
ETBF (*bft-1* gene)	F	GTACATGTTTCTATGGATAAGCGT	100
	R	CCAGTATAAAGCTGGTAGAATCC	
	P	Hex-TCCCTGCCGTTGAACAGACGGACA	
*F. nucleatum* *(nusG* gene*)*	F	CCATTACTTTAACTCTACCATGTTC	98
	R	TTGACTTTACTGAGGGAGATTATG	
	P	FAM-CAATTTCAGCAACTTGTCCTTCTTGATC	
*A. Muciniphila* *(RluA* gene*)*	F	GAAGACGGAGGACGGAACT	104
	R	CCCGGCTCTGGGATTTGAA	
	P	Hex-CTTTCCCGTATTCTGTCCGTTGAAGC	
*eae*^+^ *E. coli* *(eae* gene*)*	F	TGTAAACTATACTCCGATTCCTC	95
	R	GGAACTGCATTGAGTAAAGGAG	
	P	FAM-ACCCGTACCATGACGGTAATCGATCC	
Human *SLCO2A1* (reference gene)	F	CAGGCAGCAAGCACAGTCA	115
	R	GCAATCCCCAAAGCACCTG	
	P	Cy5-TCACAGACATTCCTGAGAGGCCAAGA	

Note: Amplicon sizes refer to the PCR products generated by the corresponding forward and reverse primer pairs; probe sequences are not included in the amplicon size.

**Table 2:** Baseline demographic, clinicopathological, and molecular characteristics of the study population.

Variables		CRC (*n* = 149)	Polyp (*n* = 109)	*p*	Adjusted *p*
Age, years, mean ± SD (range)		59.5 ± 11.6 (27—83)	58.5 ± 9.7 (31—78)	0.421	0.505
Sex, *n* (%)	Male	102 (68.5)	91 (83.5)	0.006	0.024
	Female	47 (31.5)	18 (16.5)		
Tumor location, *n* (%)	Right colon/Transverse colon	30 (20.1)	24 (22.0)	Ref	Ref
	Left colon	53 (35.6)	65 (59.6)	0.250	0.401
	Rectum	66 (44.3)	20 (18.4)	0.010	0.030
Histopathological type of polyp, *n* (%)	Adenomatous polyps	—	91 (83.5)	—	—
	Hyperplastic polyp	—	15 (13.8)	—	—
	Inflammatory polyp	—	2 (1.8)	—	—
	Peutz–Jeghers polyp	—	1 (0.9)	—	—
*BRAF* mutation*, n* (%)	*V600E*	8 (5.4)	3 (2.8)	0.365	0.487
*KRAS*, *n* (%)	Any *KRAS* mutation	55 (36.9)	23 (21.1)	0.006	0.024
*KRAS* (Exon2), *n* (%)	G12S/D	15 (10.1)	9 (8.3)	0.621	0.677
	G12C/R/V/A, G13C	22 (14.8)	11 (10.1)	0.267	0.401
	G13D	12 (8.1)	2 (1.8)	0.029	0.070
*KRAS* (Exon3), *n* (%)	A59T, Q61K	0 (0.0)	0 (0.0)		
	Q61L/R/H	5 (3.4)	1 (0.9)	0.199	0.398
*KRAS* (Exon4), *n* (%)	K117N, A146T/V/P	1 (0.7)	0 (0.0)	1.000	1.000
*HSP110*, *n* (%)	*HSP110ΔE9* transcript	26 (17.4)	41 (37.6)	<0.001	0.004

Note: Data are presented as mean ± SD or *n* (%). Continuous variables were compared using Student’s *t-*test, whereas categorical variables were compared using the chi-square test or Fisher’s exact test, as appropriate. Adjusted *p-*values were calculated using the Benjamini–Hochberg FDR procedure.

**Table 3:** Clinicopathological characteristics of patients with colorectal cancer.

Variables		CRC (*n* = 149)
Histopathology, *n* (%)	Adenocarcinoma	116 (77.9)
	Mucinous adenocarcinoma	33 (22.1)
Macroscopic type, *n* (%)	Exophytic	89 (59.7)
	Ulcer	44 (29.5)
	Ulcer-Exophytic	13 (8.7)
	Infiltration	3 (2.0)
T staging, *n* (%)	T2	10 (6.7)
	T3	40 (26.8)
	T4	99 (66.4)
N staging, *n* (%)	N0	15 (10.1)
	N1	64 (43.0)
	N2	69 (46.3)
	N3	1 (0.7)
M staging, *n* (%)	M0	134 (89.9)
	M1	15 (10.1)
Tumor grade, *n* (%)	Well differentiated	13 (8.7)
	Moderately differentiated	132 (88.6)
	Poorly differentiated	4 (2.7)

**Table 4:** Association between individual intratumoral bacterial detection and CRC.

Variable	CRC (*n* = 149) *n* (%)	Polyp (*n* = 109) *n* (%)	Crude OR (95%CI)	*p*	Adjusted OR (95%CI)	Adjusted *p*
*F. nucleatum*	102 (68.5)	51 (46.8)	2.5 (1.5–4.1)	0.001	1.37 (0.72–2.63)	0.342
ETBF	106 (71.1)	34 (31.2)	5.4 (3.2–9.3)	<0.001	4.69 (2.41–9.13)	<0.001
*A. muciniphila*	88 (59.1)	53 (48.6)	1.5 (0.9–2.5)	0.097	0.73 (0.38–1.41)	0.351
*eae^+^ E. coli*	69 (46.3)	12 (11.0)	7.0 (3.5–13.8)	<0.001	6.26 (2.92–13.42)	<0.001

Note: Crude ORs were estimated using univariable logistic regression. Adjusted ORs were estimated using a multivariable logistic regression model including all four bacterial species, age, sex, and tumor location.

**Table 5:** Bacterial co-colonization patterns associated with CRC.

Pattern	CRC (*n* = 149) *n* (%)	Polyp (*n* = 109) *n* (%)	Adjusted-OR (95%CI)	*p*	Adjusted *p*
ETBF *+ F. nucleatum*	86 (57.7)	22 (20.2)	5.70 (3.14–10.34)	<0.001	<0.001
ETBF *+ A. muciniphila*	74 (49.7)	23 (21.1)	4.11 (2.28–7.40)	<0.001	<0.001
ETBF *+ eae^+^ E. coli*	59 (39.6)	7 (6.4)	10.64 (4.49–25.24)	<0.001	<0.001
*F. nucleatum + A. muciniphila*	74 (49.7)	27 (24.8)	3.29 (1.87–5.78)	<0.001	<0.001
*F. nucleatum + eae^+^ E. coli*	52 (34.9)	7 (6.4)	8.33 (3.49–19.88)	<0.001	<0.001
*A. muciniphila + eae^+^ E. coli*	52 (34.9)	6 (5.5)	9.50 (3.82–23.60)	<0.001	<0.001
ETBF *+ F. nucleatum + A. muciniphila*	64 (43.0)	13 (11.9)	5.97 (3.00–11.86)	<0.001	<0.001
ETBF *+ F. nucleatum + eae^+^ E. coli*	43 (28.9)	3 (2.8)	14.79 (4.34–50.41)	<0.001	<0.001
*F. nucleatum + A. muciniphila + eae^+^ E. coli*	43 (28.9)	2 (1.8)	21.56 (5.02–92.60)	<0.001	<0.001
ETBF *+ A. muciniphila + eae^+^ E. coli*	42 (28.2)	4 (3.7)	10.66 (3.60–31.53)	<0.001	<0.001
ETBF *+ F. nucleatum + A. muciniphila + eae^+^ E. coli*	36 (24.2)	0 (0)	NE	NE	NE

Note : Each bacterial co-colonization pattern was evaluated using a separate multivariable logistic regression model adjusted for age, sex, and tumor location. Adjusted *p*-values were calculated using the Benjamini–Hochberg FDR procedure. NE, not estimable because no participant in the polyp group exhibited simultaneous colonization with all four bacterial species (complete separation).

**Table 6:** Association between intratumoral bacterial load and colorectal cancer.

Number of Detected Bacteria	CRC (*n* = 149) *n*, %	Polyp (*n* = 109) *n*, %	Adjusted OR (95% CI)	*p*
0	19 (12.8)	29 (26.6)	Reference	
1	15 (10.1)	32(29.4)	0.67 (0.27–1.66)	0.392
2	31 (20.8)	26 (23.9)	2.06 (0.91–4.79)	0.084
3	48 (32.2)	22 (20.2)	3.84 (1.73–8.86)	<0.001
4	36 (24.2)	0 (0.0)	112.00 (13.71–14,614.13)	<0.001
*P* for trend				<0.001

Note : Adjusted ORs were estimated using Firth’s penalized logistic regression, with bacterial load entered as a categorical variable (reference = 0 detected bacteria) and adjusted for age, sex, and tumor location. Firth’s penalized likelihood was used because complete separation occurred in the four-species category, in which 36 CRC cases (24.2%) and no polyp cases were observed. *P* for trend was obtained from a separate multivariable logistic regression model treating the number of detected bacterial species as a continuous variable.

**Table 7:** Associations between intratumoral bacterial detection and clinicopathological characteristics in CRC (*n* = 149).

Characteristics	Category	ETBF			*F. nucleatum*			*A. muciniphila*			*eae^+^ E. coli*		
		Adjusted OR (95% CI)	*p*	Adjusted *p*	Adjusted OR (95% CI)	*p*	Adjusted *p*	Adjusted OR (95% CI)	*p*	Adjusted *p*	Adjusted OR (95% CI)	*p*	Adjusted *p*
Tumor Location *n* (%)	Right/transverse colon	Reference	–	–	Reference	–	–	Reference	–	–	Reference	–	–
	Left colon	0.71 (0.27–1.90)	0.497	0.688	1.37 (0.51–3.66)	0.533	0.952	0.84 (0.33–2.13)	0.712	0.859	0.96 (0.38–2.41)	0.936	0.936
	Rectum	0.63 (0.24–1.61)	0.334	0.612	0.88 (0.34–2.31)	0.802	0.952	0.91 (0.37–2.22)	0.834	0.859	1.17 (0.49–2.84)	0.721	0.865
Histopathology *n* (%)	Adenocarcinoma	Reference	–	–	Reference	–	–	Reference	–	–	Reference	–	–
	Mucinous adenocarcinoma	1.56 (0.69–3.55)	0.290	0.612	0.74 (0.31–1.77)	0.504	0.952	1.50 (0.69–3.29)	0.306	0.673	0.55 (0.25–1.21)	0.136	0.825
Macroscopic type *n* (%)	Exophytic	Reference	–	–	Reference	–	–	Reference	–	–	Reference	–	–
	Ulcerative	1.56 (0.71–3.44)	0.267	0.612	0.66 (0.28–1.54)	0.335	0.952	0.92 (0.44–1.93)	0.822	0.859	1.47 (0.71–3.06)	0.300	0.825
	Ulcerative–exophytic	1.85 (0.55–6.28)	0.323	0.612	5.10 (1.42–18.33)	0.013	0.143	1.68 (0.52–5.45)	0.384	0.702	1.61 (0.49–5.33)	0.433	0.832
	Infiltrative	1.71 (0.14–20.26)	0.671	0.748	1.45 (0.12–17.36)	0.767	0.952	0.80 (0.07–9.36)	0.859	0.859	2.17 (0.19–25.22)	0.536	0.842
Tumor grade *n* (%)	Low	Reference	–	–	Reference	–	–	Reference	–	–	Reference	–	–
	Middle	0.43 (0.14–1.38)	0.155	0.612	0.76 (0.23–2.51)	0.657	0.952	0.41 (0.13–1.34)	0.141	0.641	0.49 (0.14–1.69)	0.261	0.825
	High	1.11 (0.12–10.66)	0.926	0.926	0.43 (0.03–5.48)	0.515	0.952	0.21 (0.02–2.70)	0.233	0.641	0.41 (0.04–4.14)	0.454	0.832
T stage *n* (%)	T1–2	Reference	–	–	Reference	–	–	Reference	–	–	Reference	–	–
	T3–4	1.32 (0.59–2.92)	0.500	0.688	1.07 (0.53–2.16)	0.853	0.952	1.31 (0.65–2.64)	0.447	0.702	1.09 (0.57–2.08)	0.786	0.865
N stage *n* (%)	N0	Reference			Reference	–	–	Reference	–	–	Reference	–	–
	N1–2	1.14 (0.62–2.10)	0.680	0.748	1.02 (0.57–1.82)	0.942	0.952	0.68 (0.40–1.17)	0.162	0.641	0.90 (0.53–1.54)	0.710	0.865
M stage *n* (%)	M0	Reference	–	–	Reference	–	–	Reference	–	–	Reference	–	–
	M1	2.29 (0.77–6.84)	0.138	0.612	0.97 (0.31–3.04)	0.952	0.952	0.48 (0.14–1.60)	0.231	0.641	0.51 (0.17–1.54)	0.232	0.825

Note: Adjusted ORs were estimated using separate multivariable logistic regression models adjusted for age and sex. Multinomial logistic regression was applied to outcomes with more than two categories (tumor location, macroscopic type, and tumor grade), whereas binary logistic regression was used for dichotomous outcomes (histopathology, T stage, N stage, and M stage). Reference categories are indicated in the table. Adjusted *p*-values were calculated using the Benjamini–Hochberg FDR procedure.

**Table 8:** Associations between bacterial detection and molecular alterations.

Characteristics	ETBF			*F. nucleatum*			*A. muciniphila*			*eae*^+^ *E. coli*		
	Adjusted OR (95% CI)	*p*	Adjusted *p*	Adjusted OR (95% CI)	*p*	Adjusted *p*	Adjusted OR (95% CI)	*p*	Adjusted *p*	Adjusted OR (95% CI)	*p*	Adjusted *p*
Any *KRAS* mutation	1.19 (0.56–2.53)	0.650	0.725	1.40 (0.66–2.96)	0.381	0.574	1.24 (0.62–2.47)	0.545	0.629	1.03 (0.52–2.03)	0.931	0.931
*KRAS* exon2	0.79 (0.36–1.72)	0.547	0.725	0.58 (0.26–1.27)	0.171	0.428	0.84 (0.41–1.71)	0.629	0.629	1.08 (0.70–1.66)	0.733	0.931
*KRAS* exon3	4.68 (0.72–30.30)	0.105	0.525	13.68 (1.38–135.89)	0.026	0.130	2.21 (0.35–14.02)	0.400	0.629	3.81 (0.41–35.62)	0.242	0.931
*KRAS* exon4	NE	NE	–	NE	NE	–	NE	NE	–	NE	NE	–
*BRAF V600E*	1.46 (0.27–7.76)	0.656	0.725	0.90 (0.20–4.08)	0.889	0.889	0.70 (0.16–2.99)	0.629	0.629	1.16 (0.27–4.95)	0.839	0.931
HSP110ΔE9 transcript	1.19 (0.45–3.12)	0.725	0.725	0.71 (0.29–1.75)	0.459	0.574	0.75 (0.32–1.77)	0.509	0.629	1.44 (0.61–3.40)	0.407	0.931

Note: Adjusted ORs were estimated using separate multivariable logistic regression models adjusted for age and sex. *p*-values were adjusted for multiple comparisons using the Benjamini–Hochberg FDR procedure. NE, not estimable due to insufficient events for model estimation.

**Table 9:** Association between bacterial detection and molecular alterations (*n* = 149).

Number of Detected Bacteria	*KRAS* Mutation			*BRAF* V600E		HSP110ΔE9 Transcript	
	Adjusted OR (95% CI)	*p*	Adjusted *p*	Adjusted OR (95% CI)	*p*	Adjusted OR (95% CI)	*p*
0	Reference	–	–	Reference	–	Reference	–
1	1.49 (0.30–7.57)	0.628	0.945	NE	NE	NE	NE
2	0.74 (0.22–2.49)	0.624	0.945	NE	NE	NE	NE
3	0.48 (0.15–1.50)	0.208	0.945	NE	NE	NE	NE
4	0.96 (0.28–3.24)	0.945	0.945	NE	NE	NE	NE
*P* for trend (per additional bacterial species)	1.01 (0.73–1.41)	0.944	0.945	NE	NE	NE	NE

Note: Adjusted ORs for each bacterial detection category were estimated using a multivariable logistic regression model with bacterial burden entered as a categorical variable (reference = 0 bacteria), adjusting for age, sex, and tumor location. *P* for trend was obtained from a separate multivariable logistic regression model treating the number of detected bacteria as a continuous variable. NE, not estimable due to insufficient events and complete separation. *p*-values were adjusted for multiple comparisons using the Benjamini–Hochberg FDR procedure.

**Table 10:** Intratumoral bacterial load according to molecular alterations in CRC (*n* = 149).

Characteristics		ETBF*-Quantity*			*F. nucleatum-Quantity*			*A. muciniphila-Quantity*			*eae^+^ E. coli* *-Quantity*		
		Median (Q1-Q3)	*p*	Adjusted *p*	Median (Q1-Q3)	*p*	Adjusted *p*	Median (Q1-Q3)	*p*	Adjusted *p*	Median (Q1-Q3)	*p*	Adjusted *p*
*KRAS* mutation	(+)	0.01 (0.00–0.10)	0.037	0.444	0.01 (0.00–0.04)	0.660	0.792	0.00 (0.00–0.01)	0.834	0.902	0.00 (0.00–0.02)	0.486	0.792
	(-)	0.00 (0.00–0.02)			0.00 (0.00–0.05)			0.00 (0.00–0.02)			0.00 (0.00–0.01)		
*BRAF V600E*	(+)	0.00 (0.00–0.00)	0.253	0.670	0.01 (0.00–0.04)	0.584	0.792	0.00 (0.00–0.00)	0.104	0.624	0.00 (0.00–0.03)	0.902	0.902
	(-)	0.00 (0.00–0.03)			0.01 (0.00–0.05)			0.00 (0.00–0.02)			0.00 (0.00–0.01)		
*HSP110ΔE9* transcript	(+)	0.01 (0.00–0.04)	0.616	0.792	0.00 (0.00–0.04)	0.616	0.792	0.00 (0.00–0.01)	0.279	0.670	0.00 (0.00–0.04)	0.198	0.670
	(-)	0.00 (0.00–0.03)			0.01 (0.00–0.05)			0.00 (0.00–0.02)			0.00 (0.00–0.01)		

Note: Continuous bacterial loads are presented as median (Q1–Q3). Comparisons between molecular alteration groups were performed using the Mann–Whitney U test because bacterial load distributions were non-normally distributed. *p*-values were adjusted for multiple comparisons across the 12 comparisons using the Benjamini–Hochberg FDR procedure.

## Data Availability

The original contributions presented in this study are included in the article/Supplementary Material. Further inquiries can be directed to the corresponding author.

## Supplementary Materials

The following supporting information can be downloaded at https://www.mdpi.com/article/10.3390/microorganisms14102325/s1: Table S1: Composition of the multiplex real-time PCR reactions; Table S2: Analytical performance characteristics of plasmid standard curves; Table S3: Mean Ct values and replicate numbers across plasmid standard concentrations.

## Abbreviations

The following abbreviations are used in this manuscript:

*A. muciniphila*	*Akkermansia muciniphila*
ARMS	Amplification Refractory Mutation System
*B. fragilis*	*Bacteroides fragilis*
CI	Confidence interval
CRC	Colorectal cancer
*E. coli*	*Escherichia coli*
ETBF	Enterotoxigenic *B. fragilis*
*F. nucleatum*	*Fusobacterium nucleatum*
FFPE	Formalin-fixed, paraffin-embedded
IQR	Interquartile range
MSI	Microsatellite instability
*NE*	*Not estimable*
OR	Odds ratio
SD	Standard deviation
WHO	World Health Organization

## References

[1] Sung H., Filho A.M., Laversanne M., Ferlay J., Siegel R.L., Soerjomataram I., Jemal A., Bray F. (2026). Global cancer statistics 2024: GLOBOCAN estimates of incidence and mortality worldwide for 34 cancers in 186 countries. CA A Cancer J. Clin..

[2] International Agency for Research on Cancer (2024). Global Cancer Observatory: Viet Nam Cancer Statistics Overview (GLOBOCAN 2022) (704). International Agency for Research on Cancer. https://gco.iarc.who.int/media/globocan/factsheets/populations/704-viet-nam-fact-sheet.pdf.

[3] Eng C., Jacome A.A., Agarwal R., Hayat M.H., Byndloss M.X., Holowatyj A.N., Bailey C., Lieu C.H. (2022). A comprehensive framework for early-onset colorectal cancer research. Lancet Oncol..

[4] Cao Y., Oh J., Xue M., Huh W.J., Wang J., Gonzalez-Hernandez J.A., Rice T.A., Martin A.L., Song D., Crawford J.M. (2022). Commensal microbiota from patients with inflammatory bowel disease produce genotoxic metabolites. Science.

[5] Shang F.M., Liu H.L. (2018). *Fusobacterium nucleatum* and colorectal cancer: A review. World J. Gastrointest. Oncol..

[6] Yang J., Wang X., Hu T., Huang H., Chen G., Jin B., Zeng G., Liu J. (2024). Entero-toxigenic *Bacteroides fragilis* contributes to intestinal barrier injury and colorectal cancer progression by mediating the BFT/STAT3/ZEB2 pathway. Cell Cycle.

[7] Gubernatorova E.O., Gorshkova E.A., Bondareva M.A., Podosokorskaya O.A., Sheynova A.D., Yakovleva A.S., Bonch-Osmolovskaya E.A., Nedospasov S.A., Kruglov A.A., Drutskaya M.S. (2023). *Akkermansia muciniphila*—Friend or foe in colorectal cancer?. Front. Immunol..

[8] Pleguezuelos-Manzano C., Puschhof J., Rosendahl Huber A., van Hoeck A., Wood H.M., Nomburg J., Gurjao C., Manders F., Dalmasso G., Stege P.B. (2020). Mutational signature in colorectal cancer caused by genotoxic pks+ *E. coli*. Nature.

[9] Iftekhar A., Berger H., Bouznad N., Heuberger J., Boccellato F., Dobrindt U., Hermeking H., Sigal M., Meyer T.F. (2021). Genomic aberrations after short-term exposure to colibactin-producing *E. coli* transform primary colon epithelial cells. Nat. Commun..

[10] Zhou Y., He H., Xu H., Li Y., Li Z., Du Y., He J., Zhou Y., Wang H., Nie Y. (2016). Association of oncogenic bacteria with colorectal cancer in South China. Oncotarget.

[11] Dai Z., Coker O.O., Nakatsu G., Wu W.K., Zhao L., Chen Z., Chan F.K., Kristiansen K., Sung J.J., Wong S.H. (2018). Multi-cohort analysis of colorectal cancer metagenome identified altered bacteria across populations and universal bacterial markers. Microbiome.

[12] Drewes J.L., White J.R., Dejea C.M., Fathi P., Iyadorai T., Vadivelu J., Roslani A.C., Wick E.C., Mongodin E.F., Loke M.F. (2017). High-resolution bacterial 16S rRNA gene profile meta-analysis and biofilm status reveal common colorectal cancer consortia. npj Biofilms Microbiomes.

[13] Dekker E., Tanis P.J., Vleugels J., Kasi P.M., Wallace M. (2019). Colorectal cancer. Lancet.

[14] Dorard C., De Thonel A., Collura A., Marisa L., Svrcek M., Lagrange A., Jego G., Wanherdrick K., Joly A.L., Buhard O. (2011). Expression of a mutant HSP110 sensitizes colorectal cancer cells to chemotherapy and improves disease prognosis. Nat. Med..

[15] Johnson D., Chee C.E., Wong W., Lam R.C., Tan I.B.H., Ma B.B. (2024). Current advances in targeted therapy for metastatic colorectal cancer–clinical translation and future directions. Cancer Treat. Rev..

[16] Greco L., Rubbino F., Ferrari C., Michela C., Grizzi F., Bonelli F., Malesci A., Mazzone M., Ricciardiello L., Laghi L. (2025). Association of *Fusobacterium nucleatum* with colorectal cancer molecular subtypes and its outcome. A systematic review. Gut Microbiome.

[17] Joo J.E., Chu Y.L., Georgeson P., Walker R., Mahmood K., Clendenning M., Meyers A.L., Como J., Joseland S., Preston S.G. (2024). Intratumoral presence of the genotoxic gut bacteria pks+ *E. coli*, Enterotoxigenic *Bacteroides fragilis*, and *Fusobacterium nucleatum* and their association with clinicopathological and molecular features of colorectal cancer. Br. J. Cancer.

[18] Osman M.A., Neoh H.M., Ab Mutalib N.S., Chin S.F., Mazlan L., Raja Ali R.A., Zakaria A.D., Ngiu C.S., Ang M.Y., Jamal R. (2021). *Parvimonas micra*, *Peptostreptococcus stomatis*, *Fusobacterium nucleatum* and *Akkermansia muciniphila* as a four-bacteria biomarker panel of colorectal cancer. Sci. Rep..

[19] Nguyen Duy T., Le Huy H., Dao Thanh Q., Ngo Thi H., Ngo Thi Minh H., Nguyen Dang M., Le Huu S., Ngo Tat T. (2024). Association between *Bacteroides fragilis* and *Fusobacterium nucleatum* infection and colorectal cancer in Vietnamese patients. Anaerobe.

[20] Senthakumaran T., Moen A.E.F., Tannæs T.M., Endres A., Brackmann S.A., Rounge T.B., Bemanian V., Tunsjø H.S. (2023). Microbial dynamics with CRC progression: A study of the mucosal microbiota at multiple sites in cancers, adenomatous polyps, and healthy controls. Eur. J. Clin. Microbiol. Infect. Dis..

[21] Amin M.B., Edge S.B., Greene F.L., Byrd D.R., Brookland R.K., Washington M.K., Gershenwald J.E., Compton C.C., Hess K.R., Sullivan D.C. (2017). AJCC Cancer Staging Manual.

[22] WHO Classification of Tumours Editorial Board (2019). Digestive System Tumours. WHO Classification of Tumours.

[23] Chouaibi S., Fertitta V., Porreca S., Njim L., Carcagnì A., Toietta G., Canettieri G., Zouari K., Mastouri M., Kadri Y. (2026). Genetic variants of the transporter SLC22A4 affect the abundance and survival of *Fusobacterium nucleatum* in colorectal cancer. Gut Microbes.

[24] Liu H., Luo Z., Luo F., Wang X., Wei H., Yu C.-Y. (2025). Intratumoral microbiota: A new perspective in cancer initiation, development, and therapy. RSC Pharm..

[25] Hunter J.G.L., Roach A., Nieves L., Islas A., Islas A., DiPalma M. (2026). The intratumoral microbiome: A review of the tumor microenvironment’s fourth axis shaping anti-tumor immunity, cancer prognosis, and therapeutic response. Front. Immunol..

[26] Zheng J., Chen H. (2024). Effects of intratumoral microbiota on tumorigenesis, anti-tumor immunity, and microbe-based cancer therapy. Front. Oncol..

[27] Jasemi S., Molicotti P., Fais M., Cossu I., Simula E.R., Sechi L.A. (2025). Biological Mechanisms of Enterotoxigenic *Bacteroides fragilis* Toxin: Linking Inflammation, Colorectal Cancer, and Clinical Implications. Toxins.

[28] Xia S., Ma L., Li H., Li Y., Yu L. (2025). Prevalence of enterotoxigenic *Bacteroides fragilis* in patients with colorectal cancer: A systematic review and meta-analysis. Front. Cell. Infect. Microbiol..

[29] de Souza J.B., de Almeida Campos L.A., Palácio S.B., Brelaz-de-Castro M.C.A., Cavalcanti I.M.F. (2024). Prevalence and implications of pKs-positive *Escherichia coli* in colorectal cancer. Life Sci..

[30] Chen B., Ramazzotti D., Heide T., Spiteri I., Fernandez-Mateos J., James C., Magnani L., Graham T.A., Sottoriva A. (2023). Contribution of pks(+) *E. coli* mutations to colorectal carcinogenesis. Nat. Commun..

[31] Rosendahl Huber A., Pleguezuelos-Manzano C., Puschhof J., Ubels J., Boot C., Saftien A., Verheul M., Trabut L.T., Groenen N., van Roosmalen M. (2024). Improved detection of colibactin-induced mutations by genotoxic *E. coli* in organoids and colorectal cancer. Cancer Cell.

[32] Jans M., Kolata M., Blancke G., D’Hondt A., Gräf C., Ciers M., Sze M., Thiran A., Petta I., Andries V. (2024). Colibactin-driven colon cancer requires adhesin-mediated epithelial binding. Nature.

[33] Mäklin T., Taira A., Arredondo-Alonso S., Shao Y., Stratton M.R., Lawley T.D., Aaltonen L.A., Corander J. (2025). Geographical variation in the incidence of colorectal cancer and urinary tract cancer is associated with population exposure to colibactin-producing *Escherichia coli*. Lancet Microbe.

[34] Queen J., Cing Z., Minsky H., Nandi A., Southward T., Ferri J., McMann M., Iyadorai T., Vadivelu J., Roslani A. (2025). *Fusobacterium nucleatum* is enriched in invasive biofilms in colorectal cancer. npj Biofilms Microbiomes.

[35] Zhao F., An R., Yu S., Ma Y., Liu W., Sheng C., Xie X., Zhang J. (2026). *Fusobacterium nucleatum* in cancer: Interactions with microbiota, tumour colonisation and cancer progression. Clin. Transl. Med..

[36] Dadgar-Zankbar L., Elahi Z., Shariati A., Khaledi A., Razavi S., Khoshbayan A. (2024). Exploring the role of *Fusobacterium nucleatum* in colorectal cancer: Implications for tumor proliferation and chemoresistance. Cell Commun. Signal..

[37] White M.T., Sears C.L. (2024). The microbial landscape of colorectal cancer. Nat. Rev. Microbiol..

[38] Long J., Wang J., Xiao C., You F., Jiang Y., Li X. (2024). Intratumoral microbiota in colorectal cancer: Focus on specific distribution and potential mechanisms. Cell Commun. Signal..

[39] Permain J., Hock B., Eglinton T., Purcell R. (2024). Functional links between the microbiome and the molecular pathways of colorectal carcinogenesis. Cancer Metastasis Rev..

[40] Cao Y., Xia H., Tan X., Shi C., Ma Y., Meng D., Zhou M., Lv Z., Wang S., Jin Y. (2024). Intratumoural microbiota: A new frontier in cancer development and therapy. Signal Transduct. Target. Ther..

[41] Zong Z., Zeng W., Li Y., Wang M., Cao Y., Cheng X., Jin Z., Mao S., Zhu X. (2024). Intratumor microbiota and colorectal cancer: Comprehensive and lucid review. Chin. J. Cancer Res..

[42] Kvich L., Fritz B.G., Zschach H., Terkelsen T., Raskov H., Høst-Rasmussen K., Jakobsen M.R., Gheorghe A.G., Gögenur I., Bjarnsholt T. (2024). Biofilms and core pathogens shape the tumor microenvironment and immune phenotype in colorectal cancer. Gut Microbes.

