# Supplementary material for: Intratumoral Bacterial Profiles, Bacterial Co-Detection Patterns, and Molecular Alterations in Colorectal Cancer and Colorectal Polyps: A Vietnamese Cohort Study
Source: Microorganisms. 2026 Oct 12;14(10):2325. doi: 10.3390/microorganisms14102325 (PMC13707697; doi:10.3390/microorganisms14102325)
Supplement: Supplementary file 1 [file microorganisms-14-02325-s001.zip › microorganisms-4582912-supplementary.pdf]

Supplementary Table S1. Composition of the multiplex real-time PCR reactions

Component	Reaction 1: *bft-1* + *nusG*	Reaction 2: *RluA* + *eae* + *SLCO2A1*
PCR-grade water (μL)	5.94	4.815
2× Reaction Mix (μL)	15.00	15.00
bft-1 forward primer, 10 μM (μL)	0.60	—
bft-1 reverse primer, 10 μM (μL)	0.60	—
nusG forward primer, 10 μM (μL)	0.60	—
nusG reverse primer, 10 μM (μL)	0.60	—
eae forward primer, 10 μM (μL)	—	0.45
eae reverse primer, 10 μM (μL)	—	0.45
RluA forward primer, 10 μM (μL)	—	0.60
RluA reverse primer, 10 μM (μL)	—	0.60
SLCO2A1 forward primer, 10 μM (μL)	—	0.60
SLCO2A1 reverse primer, 10 μM (μL)	—	0.60
bft-1 probe (HEX), 10 μM (μL)	0.30	—
nusG probe (FAM), 10 μM (μL)	0.30	—
eae probe (FAM), 10 μM (μL)	—	0.225
RluA probe (HEX), 10 μM (μL)	—	0.30
SLCO2A1 probe (Cy5), 10 μM (μL)	—	0.30
Platinum Taq (μL)	1.00	1.00
ROX dye, 25 μM (μL)	0.06	0.06
Template DNA (μL)	5.00	5.00
**Final volume (μL)**	**30.00**	**30.00**

Final concentrations: In Reaction 1, each *bft-1* and *nusG* primer was used at 200 nM and each corresponding probe at 100 nM. In Reaction 2, *eae* primers were used at 150 nM, *RluA* and *SLCO2A1* primers at 200 nM, and the corresponding probes at 75, 100, and 100 nM, respectively. Each reaction contained 150 ng of template DNA.

Thermal cycling conditions: Initial denaturation at 95°C for 2 min, followed by 45 cycles of 95°C for 30 s and 60°C for 30 s. Fluorescence was acquired at 60°C.

Supplementary Table S2. Analytical performance characteristics of plasmid standard curves

Target	Slope	Amplification efficiency (%)	R^2
bft-1 (ETBF)	−3.465	94.37	1.0000
nusG (*F. nucleatum*)	−3.445	95.12	0.9998
RluA (*A. muciniphila*)	−3.311	100.45	0.9995
eae (*eae*$^+$ *E. coli*)	−3.363	98.32	0.9995
SLCO2A1 (human internal control)	−3.309	100.55	0.9998

Abbreviations: ETBF, enterotoxigenic *Bacteroides fragilis*; R^2, coefficient of determination.

Supplementary Table S3. Mean Ct values and replicate numbers across plasmid standard concentrations

Plasmid copies/reaction	*bft-1*		*nusG*		*RluA*		*eae*		*SLCO2A1*	
	Ct, mean ± SD	n	Ct, mean ± SD	n	Ct, mean ± SD	n	Ct, mean ± SD	n	Ct, mean ± SD	n
10^6	19.272 ± 0.171	13	19.157 ± 0.405	13	18.394 ± 0.160	13	17.795 ± 0.315	13	17.668 ± 0.240	13
10^5	22.796 ± 0.208	13	22.639 ± 0.418	13	21.702 ± 0.145	13	21.074 ± 0.398	13	20.893 ± 0.233	13
10^4	26.271 ± 0.171	13	26.073 ± 0.409	13	25.188 ± 0.161	13	24.513 ± 0.455	13	24.157 ± 0.244	13
10^3	29.693 ± 0.332	13	29.480 ± 0.699	13	28.443 ± 0.337	13	27.976 ± 0.407	13	27.598 ± 0.194	13
10^2	33.193 ± 0.394	13	33.124 ± 0.611	13	31.896 ± 0.612	13	31.465 ± 0.614	13	31.029 ± 0.384	13
10^1	36.602 ± 0.454	13	36.297 ± 1.373	13	34.791 ± 0.369	13	34.408 ± 0.535	13	34.059 ± 0.203	13

Abbreviations: Ct, cycle threshold; SD, standard deviation.

Note: Values are presented as mean Ct ± SD. The 10-copy/reaction concentration represents the lowest concentration tested and detected in the analytical validation series and was not considered a formally established limit of detection (LoD) or limit of quantification (LoQ).
